# Pixel-Guided Association for Multi-Object Tracking

**DOI:** 10.3390/s22228922

**Published:** 2022-11-18

**Authors:** Abhijeet Boragule, Hyunsung Jang, Namkoo Ha, Moongu Jeon

**Affiliations:** 1 School of Electrical Engineering and Computer Science, Gwangju Institute of Science and Technology, Gwangju 61005, Republic of Korea; 2 LIG Nex1 Company Ltd., Yongin-si 16911, Republic of Korea

**Keywords:** multi-object tracking, transformer, object detection

## Abstract

Propagation and association tasks in Multi-Object Tracking (MOT) play a pivotal role in accurately linking the trajectories of moving objects. Recently, modern deep learning models have been addressing these tasks by introducing fragmented solutions for each different problem such as appearance modeling, motion modeling, and object associations. To bring unification in the MOT task, we introduce a pixel-guided approach to efficiently build the joint-detection and tracking framework for multi-object tracking. Specifically, the up-sampled multi-scale features from consecutive frames are queued to detect the object locations by using a transformer–decoder, and per-pixel distributions are utilized to compute the association matrix according to object queries. Additionally, we introduce a long-term appearance association on track features to learn the long-term association of tracks against detections to compute the similarity matrix. Finally, a similarity matrix is jointly integrated with the Byte-Tracker resulting in a state-of-the-art MOT performance. The experiments with the standard MOT15 and MOT17 benchmarks show that our approach achieves significant tracking performance.

## 1. Introduction

Multi-object tracking (MOT) has a variety of applications, including autonomous driving, sports video analysis, robot navigation, and visual surveillance. MOT helps to analyze the behavior of moving objects, and it also estimates the trajectory of moving objects. The applications of MOT are very essential in crowded places to analyze the movements of people in video surveillance systems. Recently, the performance of MOT algorithms has been significantly improved.

In the tracking-by-detection paradigm, an external detector generates the bounding box detections, and a tracker takes those detections as input for the data association task. The main objectives are to estimate the states of the moving objects and assign a unique identifier for each moving object. Multi-object tracking can be performed by online or offline processing to estimate the trajectories. Online MOT methods take the detections at the current frame and immediately generate the trajectories [1,2,3,4,5,6], whereas near-online approaches look ahead for a certain number of frames before linking to the trajectories [7,8]. In offline tracking, a mini-batch of detections is formed and processed recursively to generate the final trajectories [9,10,11,12]. Recently, many approaches have introduced a joint detection and tracking paradigm by performing end-to-end inference [13,14,15,16] on upcoming frames. These MOT methods [13,14,15,16,17] use neural network architectures for multi-object tracking and achieve state-of-the-art performance on the public MOT benchmarks.

The common assumption in joint detection and tracking methods is to use the off-the-shelf object detector and link the detected objects using a tracking head which results in higher MOT accuracy due to fewer false positives. Current ROI-based approaches fail to detect all objects when a camera scene contains small objects, and the tracking complexity increases at the tracking head when a frame contains a large number of objects. Our main goal is to improve joint detection and tracking by utilizing per-pixel distributions along with the transformer–decoder, which contributes to robust association and object center detections.

**Figure 1:**
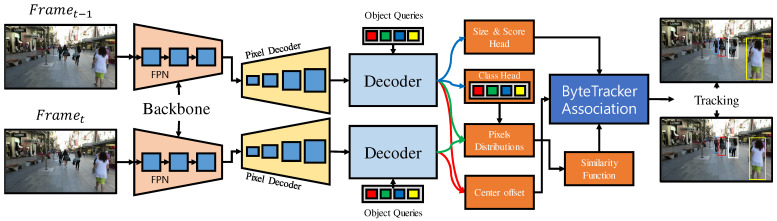
Our proposed architecture. The shared backbone is used to generate multi-scale feature embeddings. An encoder generates memory of frame multi-scale features $I_{t-1}$ and queues it to the decoder of $I_{t0}$. The encoder and decoder consist of multi-head self-attention layers, cross-attentions, and feed-forward networks. The pixel distribution between $I_{t-1}$ and $I_{t0}$ of decoder memory is generated to compute the similarity matrix. The similarity matrix is then integrated with state-of-the-art ByteTrack [23] association framework, Finally, the bounding box, class score, and center offset are generated using multi-layer perception heads. Blue arrows indicate the current frame bounding box, size, and class score. Red and green arrows indicate the consecutive frame features used for the reid association.

A key challenge is the integration of multiple models, such as appearance affinity, motion, and contextual cues for multi-object tracking. These key models are essentially propagation and association tasks. Many approaches have introduced specific and fragmented solutions for each MOT task, such as improvements in Reid networks, motion models, and different learning strategies. The transformer-based architectures have shown promising results in object detection [18,19] and multi-object tracking [13,20,21,22,22]. In the joint detection and tracking paradigm, we have introduced an end-to-end approach to propagate and associate the moving targets. The transformer-based architecture is used to generate pixel-wise distributions as the prominent Reid features within the network to track the multi-objects with a ByteTrack [23] association framework. More specifically, the consecutive frames are processed through a shared backbone network to generate the multi-scale FPN features. Afterward, the FPN features are transformed using resizing and concatenation functions. Finally, the transformed features are used in multi-scale deformable encoder–decoder. We have extracted the decoder’s last layer up-sampled pixel-wise distribution and computed the similarity matrix between detections and tracks. The resulting similarity matrix is integrated with ByteTrack [23] association. The proposed approach is shown in Figure 1.

The details of the rest of the paper are as follows. In Section 2, related works based on conventional and modern MOT approaches are discussed. In Section 3, our proposed approach and its modules are introduced. In Section 4 and Section 5, the ablation studies and MOT comparison with the other state-of-the-art methods are presented. At last, we conclude our method in Section 6.

## 2. Related Works

In multi-object tracking, an object’s similar appearance and unpredictable motion make the tracking problem more challenging. The majority of solutions have been proposed to solve these MOT challenges [2,4,5,6,9,11,13,14,17,24,25,26,27,28,29,30]. We review the conventional and transformer-based joint detection multi-object tracking frameworks.

### 2.1. Conventional Online Propagation and Association in MOT

Several Bayesian motion models have been proposed for target tracking, and they have achieved significant success in sonar-based multi-target tracking. The visual-based multi-object tracking approaches have been using Bayesian models for motion predictions, and most of the works are proposed by combining the appearance and motion models [8,31,32,33]. However, neither the appearance information nor the motion information is sufficient to perform multi-object tracking. In motion-based tracking, a simple Kalman filter [34] has been widely used for prediction and estimation. After significant success in sensor-based tracking [35], many motion-based approaches [2,3,23,31,32,36] use a Kalman filter as a basic predictor in their method. The motion models help to reduce the search space for the appearance model by predicting likelihood positions, which increases the overall speed of the tracker.

After the potential growth of the deep learning era, the majority of researchers [37,38] have been using convolution neural networks as an appearance model in MOT. Many approaches use conventional and deep appearance models for object appearance discrimination [7,15,36]. DeepSORT [17] used the bounding box overlap and deep appearance features from the neural network to associate bounding boxes with the generated tracks. In [1], to handle appearance, motion, and object interaction, three different RNNs have been modeled and trained separately for each different task. Recently, a Bilinear LSTM architecture has been used with the MHT framework [7]. This approach processing is semi-online, and it uses recurrent architecture as a regressor for the gating. The LSTM output is formulated as a least square regression, and the candidate is assigned to the tracklet based on the tracklet’s score. CenterTrack [16] predicted the previous location by using center offsets, and it reduced the post-processing overhead by jointly detecting and tracking moving objects [39,40].

### 2.2. Transformer-Based Multi-Object Tracking

Initially, a transformer model was introduced in natural language processing research. The transformer showed significant performance in computer vision tasks, such as image classification, segmentation, object detection, pose estimation, and 3D recognition. As object detection is a seminal step in the tracking, a similar end-to-end model named DETR was proposed in [18], and later it was extended to the object-centric approach by [19]. In [41], TransVOD used a temporal query encoder to aggregate the transformer output queries from different frames. Later, MOTR [21] used a vanilla transformer to update the track queries from frames by a tracking-by-attention scheme. TrackFormer [42] applied a similar approach to the MOT domain and handled newly appearing objects and tracks using a transformer. The Transcenter [13] computed the offset prediction from the previous frame and performed object associations.

Use of pixel-wise features in the vision domain has shown significant performance for different applications. The PAFormer [43] utilized a flow-guided approach by wrapping the pixel decoder features with the optical flow. The bi-linearly transformed features with an optical flow are used to detect the object inside the frame. It is noted that our approach used only up-sampled pixel distributions without optical flow. As result, it reduced extra computational overhead of our tracker.

## 3. Proposed Approach

The transformer-based architectures have shown significant improvements in detecting and tracking multiple objects in the form of bounding boxes [15] or center points [16]. Different from [13], the object locations are propagated using a transformer-based meta-architecture. Afterward, the long-term similarity function is used to associate the object with the tracks.

### 3.1. Transformer-Based Propagation

Our method uses a ResNet50 [44] to compute the multi-scale feature pyramid network features, and the transformer encoder produces the latent memory features as follows.

The consecutive image frames from the video are the input to our architecture. We process $I_{\left(t-1\right)}$ and $I_{\left(t\right)}$ through backbone architecture to generate the low resolution features $F_{t}=backbone\left(I_{\left(t\right)}\right)$. The $F_{t}\in R^{dXHxW}$ contain the height *H* and width *W* of the multi-scale features, and *d* is the dimensions. Similarly, $F_{\left(t-1\right)}$ features are extracted from the $I_{\left(t-1\right)}$ by using the same backbone network with the shared model parameters.

Our approach uses a transformer-based encoder to compute the internal latent memory between two consecutive frames. The transformer encoder consists of a multi-layer attention head which takes the backbone features $F_{\left(t\right)}$ in dXHW dimensions for each FPN layer. We supply the positional encoding, which is added with the input feature vector [19]. Inspired by the recent works [45,46], masked attention is used to deal with small objects. These attentions are useful to compute the center local representations. The encoder has computed the hidden state using multi-scale deformable DETR, which has learned the context information by self-attention. The mask-based attention [45,47] mechanism can be represented as follows,

(1) $$X_{l}=softmax\left(M_{l-1}+Q_{l}K_{l}^{T}\right),V_{l}+X_{l-1}$$

The $X_{l}\in R^{NXC}$ are the query features at the index layer *l*, and $M_{l}$ is the mask matrix and the linear transformation. The mask matrix has initialized as a zero matrix, and the linear transformation has been used as query features $Q_{l}=f_{Q}\left(X_{l-1}\right)$. The key $K_{l}=f_{K}\left(F_{t}\right)$ and value $V_{l}=f_{V}\left(F_{t}\right)$ are computed using image features under the transformation $f_{Q}\left(.\right),f_{K}\left(.\right)$ and $f_{V}\left(.\right)$. The masked attention features are passed through a standard self-attention mechanism and feed-forward network. We have propagated the last 2048 dimension channel by using bi-linear interpolations over the last FPN layer. The head for each layer output is constructed, and for each index *l*, the query embeddings are decoded by using multi-layer perceptron to obtain the object classes, object bounding boxes, and the per-pixel pixels distributions according to query features as follows,

(2) $$O_{l}^{class}=MLP^{class}\left(Q_{l}\right),$$

(3) $$O_{l}^{score}=MLP^{score}\left(Q_{l}\right),$$

(4) $$O_{l}^{center}=MLP^{center}\left(Q_{l}\right)$$

(5) $$O_{l}^{size}=MLP^{size}\left(Q_{l}\right)$$

where the MLP is multi-layer perceptron. The per-pixel distributions from the standard deformable transformer–decoder [19] is utilized to compute the pixel distribution using object center $O_{l}^{center}$ as follows,

(6) $$P_{l}\left[i,h,w\right]=sigmoid\left(F_{l}^{trs.}\left[h,w,:\right],O_{l}^{center}\left[i,:\right]\right)$$

(7) $$Feat^{k}=P_{l}\left[i\right]$$

where $P_{l}$ are the center maps of the pixels, *h* and *w* are the height and width of layer-wise feature maps; and $F_{l}^{trs.}$ per-pixel feature map from the decoder which is highlighted by the center maps $O_{l}^{center}$; and the Reid features $Feat_{k}$ are selected from the $P_{l}$ by using class *i*-th index after the post-processing for the *k*-th detection.

The association score is computed between track and detection as follows,

(8) $$Score_{T}^{k}=Euclidean\_Distance\left(Feat_{j}^{T},Feat_{t}^{k}\right)$$

where $Feat_{j}^{T}$ is the features of object *j*-th at trajectory *T*, and $Feat_{k}$ is the detected object’s features at time *t*. As our method takes two consecutive images, we process the above same operation for the $F_{\left(t-1\right)}$. The resulting object features of consecutive frames from the transformer–decoder are processed by a center offset head to propagate the object center locations for each detected target.

### 3.2. Long-Term Discriminative Appearance Matching

**Figure 2:**
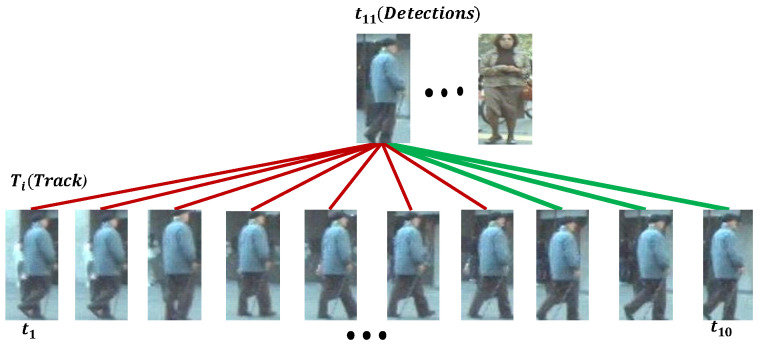
In long-term appearance matching, the comparison is conducted between $\left(t_{11},t_{1}\right),\left(t_{11},t_{2}\right),...,\left(t_{11},t_{10}\right)$ at the time of track append. The edges show the comparison between detected features and tracks. The red-colored edges are history features, and the green-colored edges are the recent features, respectively. The features of tracked and detected objects are extracted using a pixel-decoder.

We have introduced a simple yet effective long-term feature learning technique that works as a long-term association function. For each frame, the transformer–decoder generated local features for each object in an up-sampled pixel-wise distribution map. Afterward, these features are used to compute the Euclidean pairwise distance between the tracks and objects, respectively. Since we rely more on the history instead of the recent distance scores, the long-term appearance function is proposed as follows,

(9) $$App\left(T_{j},Feat_{t}^{k}\right)={\left[\frac{1}{K}\sum_{t=L-K}^{L}Score_{t}^{j}\right]}^{recent\_features}\left(1-\lambda \right)+{\left[\frac{1}{L-K}\sum_{t=1}^{L-K}Score_{t}^{j}\right]}^{history\_features}\left(\lambda \right)$$

where $T_{j}$ is a *j*-th trajectory, λ=0.90 is a learning parameter, and $Score_{t}^{j}t$ is a memory tracklet’s similarity score of the object belonging to that trajectory against the matched detection. $Feat_{t}^{k}$ are detected features at the current time *t*. The details of our proposed matching function are illustrated in Figure 2. The (L−K) represents the history length, as shown with the red color edges in Figure 2, and *K* represents the recent history length as shown with the green color edges in Figure 2. If the recent history is unreliable, then the history portion contributes to the score and vice versa. The long-term appearance module is used to produce the similarity matrix. Finally, the computed similarity matrix is used for the Byte association. In the ByteTrack algorithm, we have used a matching score m=0.65 on the similarity matrix to make non-matching candidates score zero. The track initiation and deletion threshold are used to control the track’s birth and death.

### 3.3. Training Objects

The bipartite Hungarian matching is a popular method to train the transformer-based object detectors [18,19], and its main objective is to match the ground truth objects with the predicted objects. The Mask2Former [45] used the pixel-wise distributions for the Hungarian matching. Discussed in [43], the ground truth has been modeled into Gaussian centers [16] in the heatmap, and the size of the target is modeled as the radius of Gaussian centers. The predicted heatmap by the transformer–decoder $H_{l}^{\prime }{}^{i}$ and the class distributions $C_{l}^{\prime }{}^{i}$ of *i*-th object are associated with the ground truth heatmap $H_{l}^{i}$ with the class distributions $C_{l}^{i}$. The pixel-wise cost is computed as follows,

(10) $$L_{cost}=\sum_{l}\sum_{i}\left(-\log C_{l}^{\prime }{}^{i}\left(P_{l}^{i}\right)+1_{P_{l}^{i}\neq NOB}\left|H_{l}^{\prime }{}^{i}-H_{l}^{i}\right|\right)$$

where NOB is the no-object category. To match the ground truth objects with the detected objects, the cross-entropy for the object classes, focal-loss [16] for the heatmaps, and L1 loss for the object sizes [16] are used for the architecture training. At last, we sum up all three losses and backpropagate the entire architecture for the training.

## 4. Experiments

We used the MOT15 and MOT17 datasets [48] to train our approach with public benchmarks. Afterward, the dataset was converted into training and validation sets. At the initial stage, the pretrained tracking model was used, which was trained using CrowdHuman and CityPerson object detection datasets Table 1. In our work, similar experiment protocols are followed as proposed in [13]. Finally, all datasets are converted into popular COCO style format.

### 4.1. Implementation Details

We used a popular ResNet50 [44] architecture to compute the backbone features in the form of feature pyramid networks with the dimensions of 128, 256, 512, and 2048. Next, the state-of-art object detector deformable Detr [19] was built using a multi-scale deformable transformer. The multi-scale features from the backbone architecture are processed with a multi-scale deformable transformer, which is a pixel decoder. A total of six deformable attention layers were used to generate feature maps, and we kept the resolutions the same as Mask2Former [45]. These features are then forwarded to the transformer–decoder to attain the queries with the cross attentions and feed-forward network. The layer-wise features are then extracted using object centers which map the input image bounding box locations against the feature heatmap. For each object, the channel-wise indexing is then used to generate the feature vector. We use Adam optimizer [49] to optimize our achitecture. Initially, we set the learning rate to $5\times 10^{-4}$, and adapt the scheduler learning rate with weight decay. Our model trained with 180 epochs on a single RTX 3090 GPU for six days with batch size four.

### 4.2. Metrics

We have learned the MOT metrics used by many state-of-the-art approaches for MOT performance evaluation. The meaning of each term is as follows; Multiple Object Tracking Accuracy (MOTA), Multiple Object Tracking Precision (MOTP), Correctly Identified detections (IDF1), Mostly Tracked (MT), Mostly Lost (ML), and ID Switch (IDSW).

### 4.3. Ablation Studies

**Figure 3:**
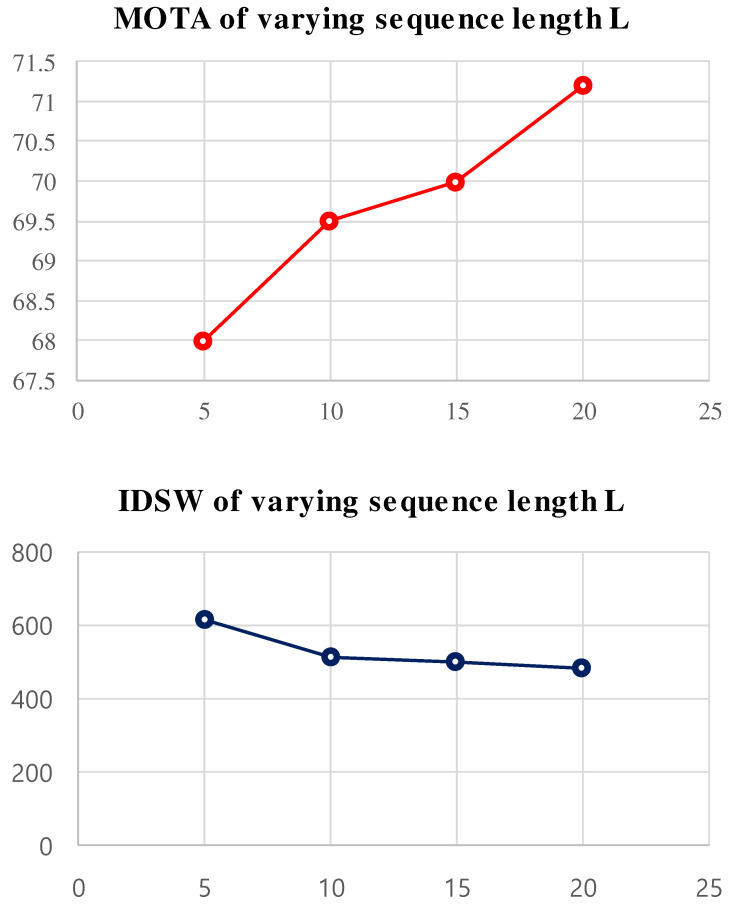
The effectiveness of our appearance model on the MOT17 training dataset for different lengths *L* and *K*.

**Discriminative appearance matching** is used to formulate the similarity matrix from the reid features. We experimented with different lengths *L* and *K* for the long-term appearance matching and showed the performance on the MOT17 training benchmark in Figure 3. In order to select a correct length, we tune the (L=5 and K=3), (L=10 and K=3), (L=15 and K=3), and (L=20 and K=3) and observe the MOT accuracy and ID switching on the dataset. In Figure 3, we have demonstrated the performance of different lengths on the MOT15 training dataset. We observed the ID switching and MOT accuracy metrics to choose a correct L=15 and K=3.

In our experiments, we set the track initiation length the same as ByteTracker [23]. In the proposed tracker, the Kalman’s constant linear velocity model [34] is used as a motion predictor. A simple Kalman motion model is independently created for each track, which predicts and updates the motion of objects. The λ=0.90 is the update ratio of appearance similarity cost. We have chosen K=3 because K=1 makes the target’s recent history unreliable when an object starts to occlude or there is a sudden appearance change.

**Figure 4:**
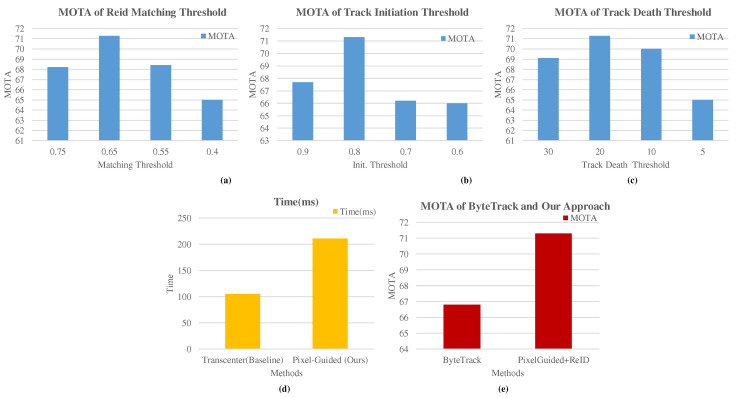
Effectiveness of our method on the MOT17 Training dataset: (**a**) MOTA of matching thresholds in Reid function; (**b**) effective track initiation thresholds on MOTA; (**c**) effective track death thresholds on MOTA; (**d**) average inference time; (**e**) effectiveness of Reid features.

**Matching threshold** is used to assign the object to tracks in the association matrix. The association matrix cells represent the association between tracks and detected objects after using a threshold on the similarity matrix. We demonstrate the effectiveness of our approach on the matching thresholds in Figure 4a, and threshold 0.65 achieved the highest MOTA on the MOT17 training dataset.

**Track initiation** was used to generate the initial track by comparing the initial object score, which is the starting point of any arbitrary track. This initiation procedure tackles the track fragmentation and avoids false positives. We have reported track matching threshold in Figure 4b, and 0.8 threshold shows better MOTA performance.

Similarly, the track death flag is enabled when an object disappears longer in the upcoming frames. In Figure 4c, 20 thresholds show better MOTA performance, and our approach waits longer for an object to reappear in the camera scene.

**Inference Time** Our approach held memory features of 20 frames for each track, and it contributed towards higher inference time. Due to the larger number of model parameters, it is challenging to speed up the entire architecture. The inference time is shown in Figure 4d.

**ReID features in ByteTrack** The Reid features from the class index are mapped into pixel-wise distribution from the last layer of the transformer–decoder. These features are used to compare the similarity between tracks and detected objects by using Equation (9). These features represent uniqueness for each object where no special metric learning is required during the training phase. The effectiveness of Reid features in ByteTrack association is shown in Figure 4e. The MOTA significantly improved after integrating the Reid-based association matrix with the ByteTrack algorithm.

## 5. Experiments with Public MOT Benchmarks

In this section, we demonstrate the performance of our approach on MOT15 and MOT17 public datasets. To compare quantitative results, we have chosen state-of-the-art trackers from the MOT challenge benchmark. In Table 2 and Table 3, we have used short names of the trackers and have partitioned them into private and public detection categories. Public detections are used to maintain the fairness of benchmark protocols.

### Comparison with the Baseline Approach

From the baseline to our method, we utilized a joint-detection and tracking framework. To check whether our trained model works well with the pixel distribution from the transformer–decoder, we have shown the comparison including the most recent state-of-the-art trackers in Table 2 and Table 3. The entire MOT paradigm is shifting towards the joint-detection and tracking paradigm, where a detector is playing the pivotal role in efficiently localizing the objects, and association methods are functioning as tracking heads. Our pixel-based distribution head utilizes deep features from the transformer–decoder. For a fair comparison, we have used the detection queries and mapped against the object queries to evaluate our approach on public detections.

**MOT 15** In Table 2, our tracker has achieved 40.6 MOTA, 51.1 IDF1, and 1129 IDsw. We have achieved excellent MT and ML measures due to the center object propagation ability of our tracker against the other SOTA trackers. The MFTST [52] has achieved higher MOTA which is currently the SOTA method for online tracking with public detections. The GSDT [39], FairMOT [14], Tube_TK [50], and RAR15 [51] achieve significantly higher performance due to private object detectors, and it proves that the tracking performance is dependant on detection quality. The private detection alignment is used with the public detections for a fair comparison by converting detection queries as object queries. Our method achieved better performance at MT, ML, and FP measures.

**Figure 5:**
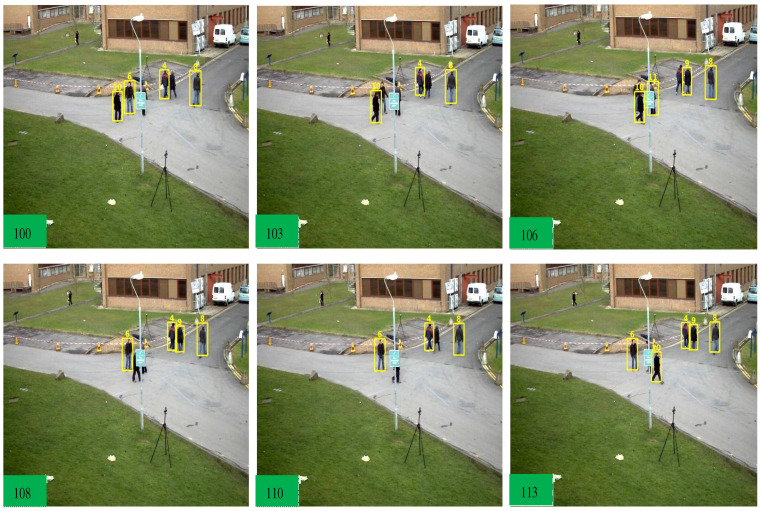
Effectiveness of our tracker in the occlusion case-1. As from the above frames, an object with the ID (10) is passing behind the pole and from frame number 103 to 110, it has no detection response due to occlusion. Our tracker re-assigns that object with the same ID (10) at frame 113.

**Figure 6:**
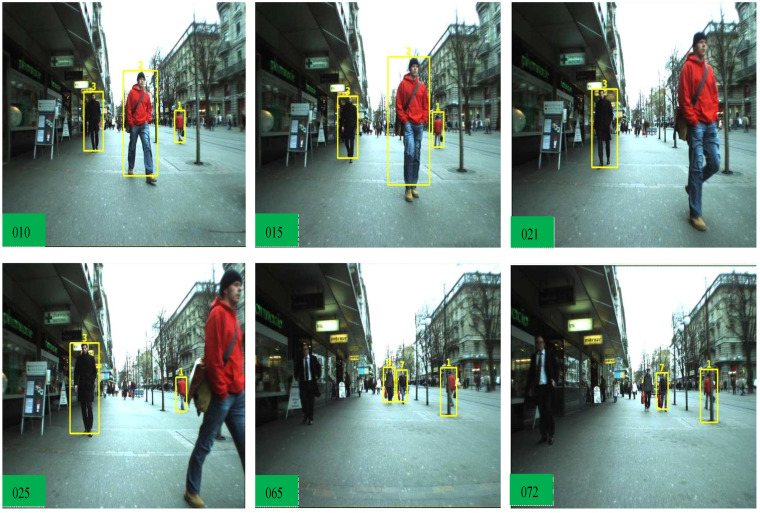
Effectiveness of our tracker in the occlusion case-2. In this case, moving objects are getting far and close to the camera view. An object with the ID (1), moving away from the camera view along with the occlusion by another object from frames 17 to 24. Our tracker re-assigns that object with the same ID (1) at frame 25. Due to the long-term appearance method, it shows that our tracker is consistent at ID keeping.

**Figure 7:**
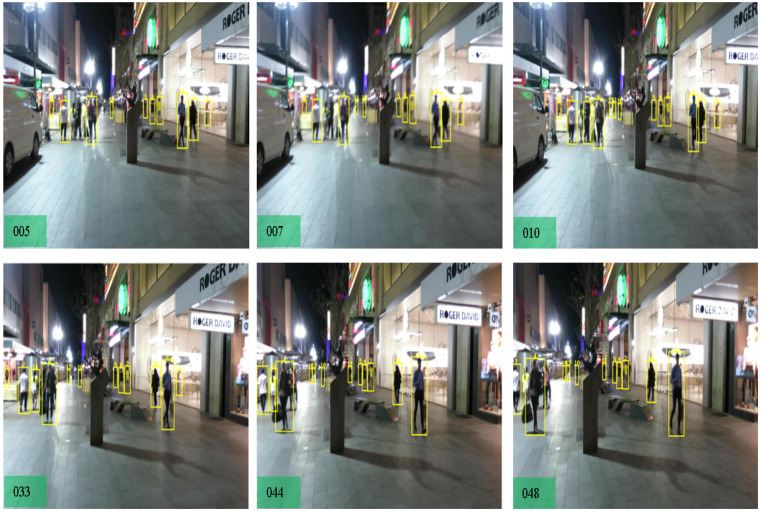
Effectiveness of our tracker in the case of moving camera. Our tracker is effectively tracking the multi-objects without becoming drifted on anchors by camera motion with the small objects inside the scene. The detected bounding box and the unique ID are demonstrated in yellow color.

**MOT 17** We compared our results with the SOTA trackers, CenterTrack [16], LSST17 [59], Tracktor [15], SiamMOT [58], QuasiDense [6], TransCenter [13], and ByteTrack [23]. Compared to the LSST17 method, our tracker surpasses the MOTA by 17% due to higher MT, lower ML, and lower IDSW measures. We then compare our method with the Tracktor [15] which is joint detection and tracking framework. The Tracktor is heavily dependent on a private object detector which is lower in MT objects, and we achieve +16.2 % MOTA. Compared to our baseline TransCenter [13], we achieve higher MOTA performance as it utilizes entire memory-based queuing within the encoder and decoders, and our pixel distribution has contributed towards lower MT, FP, and FN against TransCenter. We surpass the ByteTrack in MOTA, MT, and ML due to the detection of small objects where the ByteTrack association method works with state-of-the-art detectors. The CenterTrack and TransCenter detect center position with center offset, which does not use Reid features from the pixel distribution. After combining pixel-wise distribution features with the ByteTrack association, we achieved higher MOTA performance. The output frame samples are shown in Figure 5, Figure 6 and Figure 7 to show the effectiveness of our tracker.

**Strength and Limitations** To focus on the strength of our approach, we used the deformable DETR [19] and pixel-wise distribution as a Reid feature. More specifically, the prominent backbone FPN features are utilized to resolve the limited spatial resolution which is a key problem in standard DETR [18]. The object-centric attention over multi-scale features contributes to detecting small objects in the camera frame. As a result, our approach shows lower MT and ML performance in both MOT17 and MOT15 benchmarks. Furthermore, the pixel-wise distribution from the center heat map is mapped using class index 7. These features represent each object’s embedding which is used to compute the association matrix between tracks and objects. Table 3 shows how our approach surpasses ByteTrack in MOTA performance.

Due to a large number of model parameters and complex transformer-based architecture, our approach takes a second to process five frames which is far from real-time processing. Another downside of our approach is the multi-stage training procedures for the different datasets. Our approach uses an off-the-shelf private object detector named [19] modules where large-scale pretraining is required, and it is challenging to directly apply to MOT application. Due to the inductive biases of the pretrained modules, it is difficult to detect all the small objects of unknown domains.

## 6. Conclusions and Future Work

In this paper, we have presented the pixel-guided MOT approach, which uses transformer-based architecture to extract the pixel-wise up-sampled features for multi-object tracking. First, the consecutive frames are passed to build FPN features. Second, the robust local representation in the deformable transformer encoder–decoder is used to detect small objects in the joint detection and tracking framework. Third, the up-sampled pixel-wise features from the transformer–decoder are used to build Reid appearance features. Finally, the Reid features are integrated with a long-term appearance learning function to compute the similarity matrix. The similarity matrix is integrated with the ByteTrack association framework, which results in better MOT performance. The extensive experiments on standard MOT benchmarks have shown the effectiveness of our pixel-guided approach for multi-object tracking.

Our per-pixel distribution can extend to multi-object segmentation tasks. The transformer-based architecture can be integrated into the Yolo and faster RCNN backbone features, which can improve the performance of MOT algorithms. We will also study the lightweight transformer architectures to maintain speed and accuracy tradeoffs of MOT algorithm.

## Figures and Tables

**Table 1:** Sequences mentioned in the table are used to train the deep neural network.

Sequences	Training	Validation
MOT15 Sequences{1,2,3,4,5,6,7,10,11}	50%	50%
MOT17 Sequences{2,4,5,9,10,11,13}	50%	50%
CityScapes	16 Sequences	5 Sequences
Crowdhuman	15,000 Frames	4370 Frames

**Table 2:** Performance of our tracker on the MOT15 benchmark. Our proposed approach is in bold text.

Tracker	MOTA%↑	IDF1%↑	MT%↑	ML%↓	FP↓	FN↓	IDSW↓
Private Detector							
GSDT [39]	60.7	64.6	47.0	10.5	7334	16,358	477
FairMOT [14]	60.6	64.7	47.6	11.0	7854	15,785	591
Tube_TK [50]	58.4	53.1	39.3	18.0	5756	18,961	854
RAR15 [51]	56.5	61.0	45.1	14.6	9386	16,921	428
Public Detector							
MFI_TST [52]	**49.2**	**52.4**	210	176	8707	21,594	912
GNNMATCH [53]	46.7	43.2	157	203	6643	25,311	820
KCF [54]	38.9	44.5	120	227	7321	29,501	**720**
TrctrD15 [55]	44.1	46.0	124	192	**6085**	26,917	1347
**Pixel-Guided**	40.6	51.9	**294**	**86**	15,027	**17,352**	1129

**Table 3:** Performance of our tracker on the MOT17 benchmark. Our proposed approach is in bold text.

Tracker	MOTA%↑	IDF1%↑	MT%↑	ML%↓	FP↓	FN↓	IDSW↓
Private Detector							
FairMOT [14]	73.7	72.3	19.5	36.6	12,201	248,047	2072
PermaTrack [56]	73.8	68.9	43.8	17.2	28,998	115,104	3699
CorrTracker [57]	76.5	73.6	47.6	12.7	29,808	99,510	3369
ByteTrack [23]	80.3	77.3	53.2	14.5	25,491	83,721	2196
Public Detector							
SiamMOT [58]	65.9	63.3	34.6	23.9	14,076	200,672	2583
CenterTrack [16]	67.8	64.7	34.6	24.6	18,498	160,332	3039
QuasiDense [6]	68.7	66.3	40.6	21.9	26,589	146,643	3378
LSST17 [59]	52.7	57.9	421	863	22,512	241,936	2167
Tracktor [15]	53.5	52.3	459	861	12,201	248,047	2072
TransCtr [13]	68.8	61.4	867	**564**	22,860	149,188	4102
ByteTrack [23]	67.4	**70.0**	730	735	**9939**	172,636	**1331**
**Pixel-Guided**	**69.7**	68.4	**903**	615	26,871	**140,457**	3639

## Data Availability

Not applicable.
